# Multicenter, randomized controlled, open label evaluation of the efficacy and safety of arbidol hydrochloride tablets in the treatment of influenza-like cases

**DOI:** 10.1186/s12879-023-08570-9

**Published:** 2023-09-06

**Authors:** Xinfeng Bai, Suya Xi, Guiyan Chen, Xiaoying Fan, Kaiwei Wang, Yong Li, Yang Zhao, Weizhan Wang, Yingping Tian

**Affiliations:** 1 Emergency Department, Second Hospital of Hebei Medical University, No. 215 Heping West Road, Xinhua District, Shijiazhuang, 050051 Hebei China; 2 Hebei Chest Hospital, Shijiazhuang, Hebei China; 3 Chengde Central Hospital, Chengde, Hebei China; 4 Handan Central Hospital, Handan, Hebei China; 5 The Sixth People’s Hospital of Hengshui, Hengshui, Hebei China; 6 Cangzhou Central Hospital, Cangzhou, Hebei China; 7 Qinhuangdao Traditional Chinese Medicine Hospital, Qinhuangdao, Hebei China; 8 Harrison International Peace Hospital, Hengshui, Hebei China

**Keywords:** Arbidol hydrochloride tablets, Influenza-like cases, Multicenter study, Randomized controlled trial

## Abstract

**Objective:**

To study the efficacy and safety of arbidol hydrochloride tablets as a treatment for influenza-like diseases.

**Methods:**

In this multicenter, randomized, controlled, open label study, a total of 412 influenza-like cases were collected from 14 hospitals in seven regions of Hebei Province from September 2021 to March 2022. Patients were randomly divided into two groups. The control group (n = 207) were administered oseltamivir phosphate capsules for five days and the experimental group (n = 205) were administered arbidol hydrochloride tablets for five days. The primary endpoint was the time to normal body temperature, and the secondary endpoints included the time to remission of influenza symptoms, incidence of influenza-like complications, and incidence of adverse reactions.

**Results:**

Before treatment, there was no significant difference between the two groups in general conditions, blood routine, body temperature, or symptom severity. After treatment, there was no significant difference between the groups in the mean time to fever remission (59.24 h ± 25.21 vs. 61.05 h ± 29.47) or the mean time to remission of influenza symptoms (57.31 h ± 30.19 vs. 62.02 h ± 32.08). Survival analyses using Log-rank and Wilcoxon bilateral tests showed that there was no significant difference in fever relief time or influenza symptom relief time between the two groups. Regarding the incidence of complications and adverse events, there was only one case of tracheitis, one case of nausea, one case of vomiting, and one case of dizziness in the control group. In the experimental group, there was one case of nausea, one case of vomiting, and one case of drowsiness. In addition, one patient in the control group was hospitalized for urinary calculi.

**Conclusion:**

There was no significant difference between the patients with influenza-like cases treated with arbidol hydrochloride tablets and those treated with oseltamivir phosphate capsules. Further, the patients treated with arbidol hydrochloride tablets had fewer adverse reactions, and thus, the tablets were safe to use.

## Introduction

Influenza is an acute respiratory infectious disease caused by the influenza virus, which can cause seasonal epidemics or even pandemics, and threatens human health [1]. The influenza virus can cause respiratory diseases, encephalitis, myelitis, myocarditis, and even septic shock, resulting in a large number of severe cases and deaths and causing serious disease, social, and economic burden [2].

Arbidol hydrochloride tablets and oseltamivir phosphate are commonly used for the clinical treatment of influenza. At present, oseltamivir is the gold standard and the most widely used anti-influenza drug [3–5]. As drug-resistant variants continue to emerge naturally and through selective pressure caused by antiviral drug use, the efficacy of oseltamivir may wane over time [6–9]. Arbidol exerts substantial antiviral effects in various animal models of infection and has been used with effect in clinical trials for the prevention and treatment of influenza [10].

Arbidol has a low rate of generation of resistant strains of influenza with respect to adamantane and neuraminidase inhibitors [11, 12]. Some studies have demonstrated that oseltamivir-resistant viruses are susceptible to arbidol [13, 14], suggesting that arbidol may be a good alternative for the clinical treatment of infections caused by oseltamivir-resistant viruses. This study evaluated the efficacy and safety of arbidol in clinically diagnosed suspected influenza cases [15].

## Materials and methods

### Research participants

**Fig. 1:**
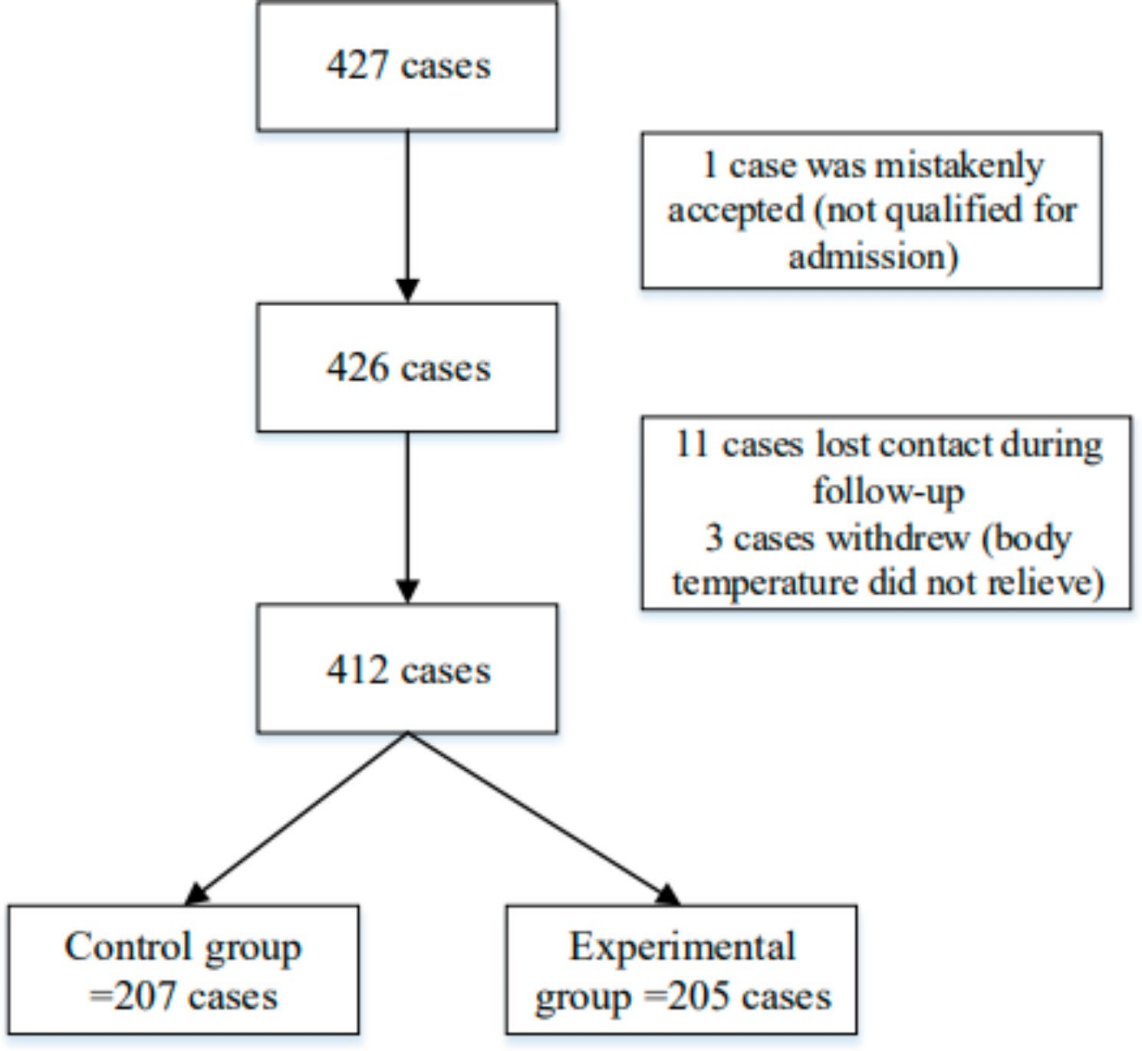
Flow chart of enrolled influenza-like cases

To further explore the effect of arbidol hydrochloride in the treatment of influenza-like cases, a total of 427 subjects meeting the clinical diagnosis criteria of influenza in the draft Guidelines for Clinical Diagnosis and Treatment of Influenza (draft) [15] of the Respiratory Department of the Chinese Medical Association were screened for this study. Among these, one individual was mistakenly accepted (not qualified for admission), 11 individuals were lost (lost contact) to follow-up, and three individuals withdrew (body temperature was not relieved), leaving a total of 412 cases for the analysis. Patients were randomly divided into two groups: 207 in the control group and 205 in the experimental group (Fig. 1). The outpatients were from the Second Hospital of Hebei Medical University, the Sixth People’s Hospital of Hengshui, the First Hospital of Hebei Medical University, Shijiazhuang Hospital of Traditional Chinese Medicine, Qinhuangdao Traditional Chinese Medicine Hospital, Chengde Central Hospital, Xingtai First Hospital, Cangzhou Central Hospital, Harrison International Peace Hospital, Qinhuangdao First Hospital, Handan Central Hospital, Hebei Chest Hospital, Handan First Hospital, and Hebei Hospital of Traditional Chinese Medicine.

The registration number is ChiCTR2100043928, and the date of registration was 5/3/2021.

The criteria for inclusion and exclusion were developed based on previous multicenter studies of oseltamivir and arbidol [16, 17].

Inclusion criteria: (1) signed informed consent; (2) age ≥ 18 years; (3) body temperature ≥ 37.5 °C, and (4) influenza-like symptoms: stuffy nose, sore throat, cough, muscle aches, fatigue, headache, chills/sweating, etc., with no diagnosis other than upper respiratory tract infection.

Exclusion criteria: (1) patients with allergic history and/or severe allergic constitution; (2) severe liver and kidney dysfunction; (3) high likelihood of bacterial infection based on signs, symptoms, or laboratory tests; (4) development of pulmonary exudative lesions; (5) severe heart disease that researchers believe will affect the safety of subjects or clinically significant arrhythmia (according to ECG or medical history); (6) influenza vaccine taken within 12 months; (7) known HIV infection; (8) pregnant or lactating women; and (9) laboratory-confirmed COVID-19.

### Methods

Methods were developed with reference to previous multicenter studies of oseltamivir and arbidol [16, 17]. This study was a randomized, controlled, open label, multicenter clinical trial. The patients’ medical history, physical examination, and routine blood test results were collected. Subjects who met the criteria were randomly divided into two groups using the block randomization method into the control group and the test group using SAS9.4 software to generate random sequences.

#### Dose regimens

Control group: oseltamivir phosphate capsules (Yichang HEC Changjiang Pharmaceutical Co., Ltd), 75 mg once, were administered twice a day for five consecutive days; experimental group: arbidol hydrochloride tablets (CSPC Ouyi Pharmaceutical Co., Ltd), 200 mg, were administered three times a day, for five consecutive days.

#### The age, sex, detailed medical history, past history, and allergy history of the patients were collected at the time of enrollment as were measurements of their vital signs and routine blood tests

The effect of treatment was assessed using log cards. Patients completed the influenza-like symptom scale (ISS) twice a day in the morning and evening, which was divided into four levels (0 asymptomatic, 1 mild, 2 moderate, and 3 severe) according to the different degrees of ISS. When the symptom was zero to one for 24 h, it was recorded as normal, and recording was stopped.

Body temperature was measured four times in the morning, noon, afternoon, and evening of every day. If the subject’s body temperature dropped below 37.3 °C for 24 h, body temperature recording was stopped.

During the visit, it was necessary to ask and record the daily use of the experimental drugs and the combined use of other drugs taken at the same time. In this study, other drugs with antiviral effects and traditional Chinese medicine preparations to treat cold, relieve cough or resolve phlegm, interferon, and glucocorticoids were prohibited. During the medication period, if other symptoms or the current flu symptoms were aggravated, the patient returned to the test unit for relevant tests. The researcher judged whether there are any complications and recorded them at that time.

#### Efficacy indicators

(1) Time to fever relief (the time, after medication, for the axillary body temperature to drop to < 37.3 °C for 24 h). Time to relief of influenza symptoms: nasal congestion, sore throat, cough, muscle soreness, fatigue, headache, chills, sweating, and other influenza symptoms were relieved (ISS score was reduced to 0 or 1), and the time required for remission to last for at least 24 h. (2) Disease severity: The area under the curve (AUC) of the total score of influenza symptoms was used as the evaluation index, and the total score of influenza symptoms twice a day was plotted with the median time (h) to calculate the AUC, and the difference between the two groups in the total symptoms score was counted. (3) The incidence of influenza-like complications (sinusitis, otitis media, myocarditis, pneumonia) and adverse events.

This study was approved by the Scientific Research Ethics Committee of the Second Hospital of Hebei Medical University (No.2020-C044-X01).

### Statistical methods

SAS9.4 and SPSS25.0 software were used to analyze the data. Categorical variables are expressed as cases (%), and the comparison between groups adopted χ^2^ inspection. Measurement variables with a normal distribution were expressed as mean ± standard deviation (x ± s). The independent sample t-test was used for comparisons between groups. Measurement variables that did not conform to a normal distribution were represented by the median and quartile-m (p25 ~ p75), and the independent sample nonparametric test was used for the comparison between groups. Statistical significance was set at P < 0.05. A survival curve method was used to analyze fever and seven clinical symptoms in each group, the Kaplan–Meier diagram was drawn, and the log-rank and Wilcoxon methods were used to test the significance of both sides.

## Results

### Comparison between the experimental group and the control group before treatment

There were no significant differences in age, sex, vital signs, or basic diseases between the two groups (Table 1). Before treatment, there was no significant difference between the two groups in white blood cell count, absolute neutrophil value, absolute lymphocyte count, red blood cell count, hemoglobin level, or platelet count (*P* > 0.05) (Table 1). There was no statistical significance between the two groups in body temperature or flu symptoms, such as headache, stuffy nose, sore throat, cough, fatigue, muscle soreness, and chills/sweating before treatment (*P* > 0.05) (Table 1).

**Table 1:** Comparison between the experimental group and the control group before treatment

	Description	Control group (n = 207)	Experimental group (n = 205)	Statistic	P-value
Gender	Male	105 (50.7%)	112 (54.6%)	0.631 (χ2)	0.427
	Female	102 (49.3%)	93 (45.4%)		
Age	Median	33.00	33.00	0.309 (Z)	0.757
	P25 ~ P75	24.00 ~ 43.00	25.00 ~ 41.00		
	Min ~ Max	18 ~ 87	18 ~ 85		
Pulse (times /min)	Median	90.00	92.00	0.501 (Z)	0.616
	P25 ~ P75	85.00 ~ 102.00	85.00 ~ 102.00		
	Min ~ Max	65 ~ 130	64 ~ 146		
Respiration rate (times /min)	Median	20.00	20.00	0.040 (Z)	0.968
	P25 ~ P75	18.00 ~ 21.00	18.00 ~ 21.00		
	Min ~ Max	14 ~ 37	12 ~ 30		
Systolic pressure (mmHg)	Median	120.00	121.00	0.503 (Z)	0.615
	P25 ~ P75	110.00 ~ 129.00	112.00 ~ 128.00		
	Min ~ Max	70 ~ 168	65 ~ 161		
Diastolic pressure (mmHg)	Median	78.00	79.00	0.240 (Z)	0.810
	P25 ~ P75	71.00 ~ 86.00	72.50 ~ 87.00		
	Min ~ Max	48 ~ 130	54–145		
**Basic diseases**					
Hypertension	Yes	6 (2.9%)	5 (2.4%)	0.084 (χ2)	0.772
	No	201 (97.1%)	200 (97.6%)		
Diabetes	Yes	3 (1.4%)	1 (0.5%).	0.990 (χ2)	0.320
	No	201 (98.6%)	204 (99.5%)		
Coronary heart disease (CHD)	Yes	1 (0.5%).	1 (0.5%).	0.001 (χ2)	0.995
	No	206 (99.5%)	204 (99.5%)		
Interstitial pneumonia	Yes	0 (0.0%)	1 (0.5%).	1.012 (χ2)	0.314
	No	201 (100.0%)	204 (99.5%)		
Chronic rhinitis	Yes	0 (0.0%)	3 (1.5%)	3.051 (χ2)	0.081
	No	201 (100.0%)	204 (98.5%)		
**Blood routine tests**					
Red blood cell count (10^12^/L)	Median	4.73	4.69	0.259(Z)	0.796
	P25 ~ P75	4.31 ~ 5.07	4.37 ~ 5.08		
	Min ~ Max	1.07 ~ 6.10	2.40 ~ 6.33		
White blood cell count (10^9^/L)	Median	6.04	5.75	0.913(Z)	0.361
	P25 ~ P75	4.60 ~ 7.40	4.60 ~ 7.00		
	Min ~ Max	1.79 ~ 11.20	2.30 ~ 16.27		
Platelet count (10^9^/L)	Median	199.50	206.00	0.955(Z)	0.340
	P25 ~ P75	160.50 ~ 244.00	169.00 ~ 249.00		
	Min ~ Max	53.00 ~ 454.00	71.00 ~ 553.00		
Hemoglobin(g/L)	Median	141.50	142.00	0.809(Z)	0.419
	P25 ~ P75	128.75 ~ 154.00	133.00 ~ 154.00		
	Min ~ Max	42.00 ~ 182.00	66.00 ~ 187.00		
Neutrophil absolute value (10^9^/L)	Median	3.99	3.76	0.393(Z)	0.695
	P25 ~ P75	2.95 ~ 5.17	2.84 ~ 5.03		
	Min ~ Max	0.40 ~ 9.54	1.10 ~ 14.36		
Lymphocyte absolute value (10^9^/L)	Median	1.08	1.09	0.042(Z)	0.967
	P25 ~ P75	0.78 ~ 1.59	0.78 ~ 1.56		
	Min ~ Max	0.35 ~ 3.84	0.31 ~ 5.50		
**Body temperature (°C)**	Median	38.30	38.20	0.743 (Z)	0.457
	P25 ~ P75	37.90 ~ 38.60	37.80 ~ 38.60		
	Min ~ Max	37.50 ~ 39.80	37.50 ~ 40.70		
**Severity of symptoms**					
Stuffy nose	No	82 (39.6%)	86 (42.0%)	2.396 (χ2)	0.494
	Light	43 (20.8%).	47 (22.9%).		
	Middle	48 (23.2%).	49 (23.9%).		
	Heavy	34 (16.4%).	23 (11.2%).		
Sore throat	No	54 (26.1%).	57 (27.8%).	3.839 (χ2)	0.279
	Light	41 (19.8%).	49 (23.9%).		
	Middle	67 (32.4%)	49 (23.9%).		
	Heavy	45 (21.7%).	50 (24.4%).		
Cough	No	70 (33.8%)	74 (36.1%)	1.852 (χ2)	0.604
	Light	58 (28.0%).	50 (24.4%).		
	Middle	56 (27.1%).	51 (24.9%).		
	Heavy	23 (11.1%).	30 (14.6%)		
Muscle soreness	No	56 (27.1%).	65 (31.7%)	3.481 (χ2)	0.323
	Light	54 (26.1%).	49 (23.9%).		
	Middle	65 (31.4%)	51 (24.9%).		
	Heavy	32 (15.5%).	40 (19.5%).		
Fatigue	No	45 (21.7%).	44 (21.5%).	6.165 (χ2)	0.104
	Light	55 (26.6%).	59 (28.8%).		
	Middle	78 (37.7%)	58 (28.3%).		
	Heavy	29 (14.0%).	44 (21.5%).		
Headache	No	62 (30.0%)	68 (33.2%)	4.024 (χ2)	0.259
	Light	48 (23.2%).	50 (24.4%).		
	Middle	68 (32.9%)	50 (24.4%).		
	Heavy	29 (14.0%).	37 (18.0%)		
Chills/sweating	No	65 (31.4%)	68 (33.2%)	7.136 (χ2)	0.068
	Light	48 (23.2%).	55 (26.8%).		
	Middle	68 (32.9%)	45 (22.0%).		
	Heavy	26 (12.6%).	37 (18.0%)		

### Comparison of efficacy between the two groups

#### Comparison of fever relief time and influenza symptom relief time

**Fig. 2:**
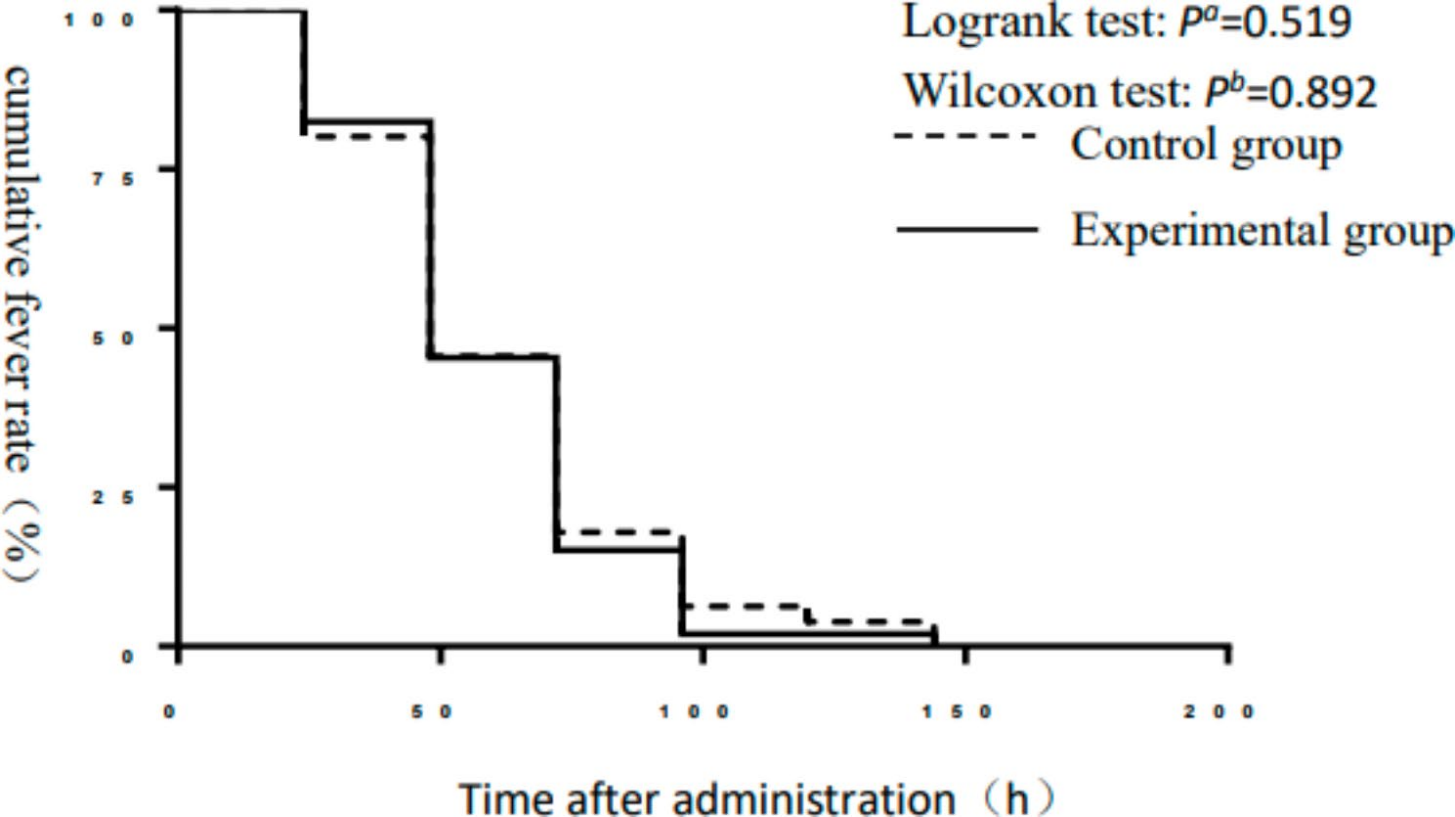
Cumulative fever rate survival curves of the control and experimental groups

**Fig. 3:**
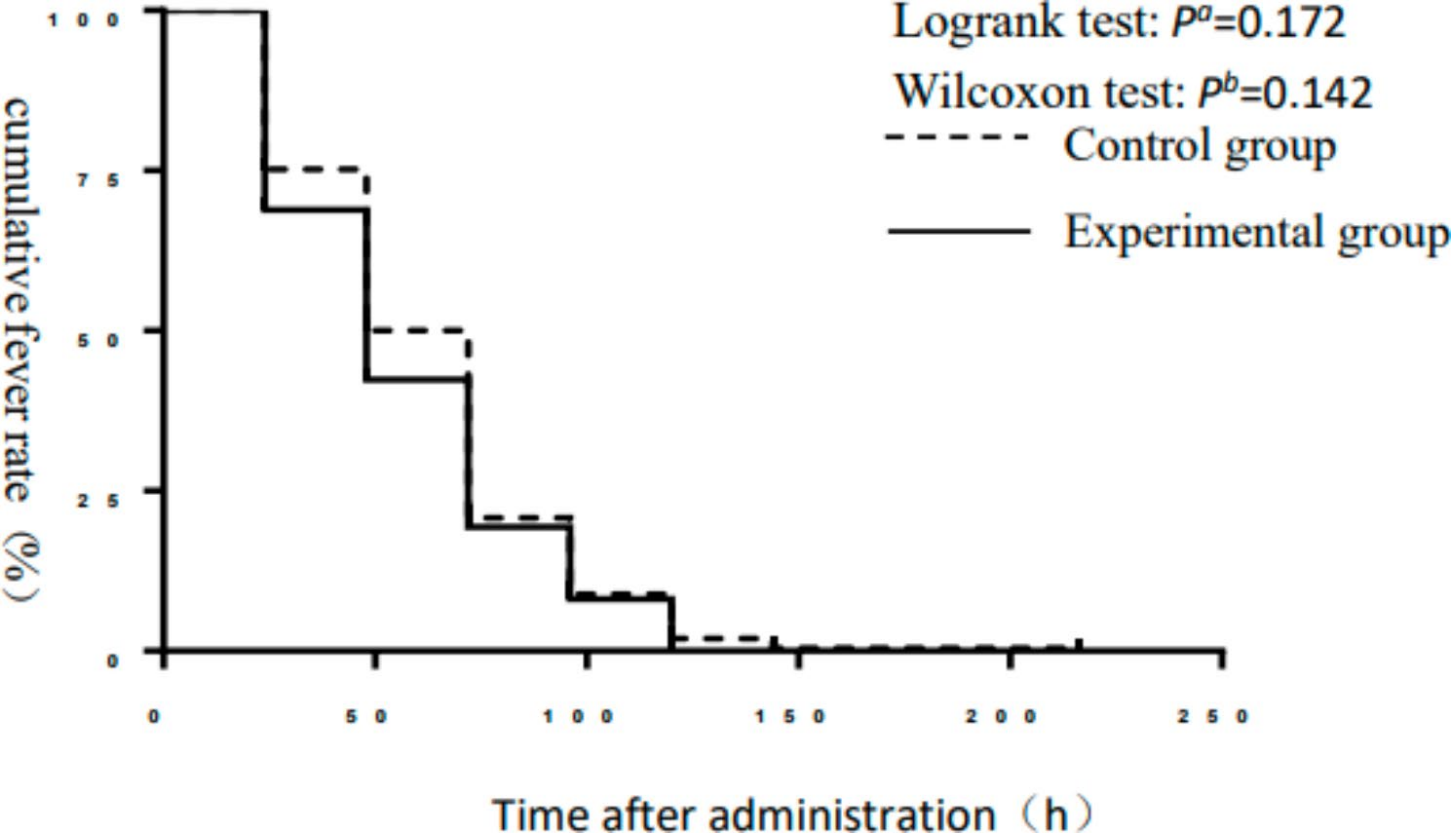
Survival curves of the cumulative influenza symptom ratio in the control and experimental groups

There was no significant difference in time to fever relief or influenza symptom relief between the experimental and control groups (*P* > 0.05). Log-rank and Wilcoxon bilateral tests showed that there was no significant difference in the survival curve analysis of time to fever relief time or influenza symptom relief between the two groups (*P* > 0.05) (Table 2; Figs. 2 and 3).

**Table 2:** Time to body temperature and symptom relief in the experimental and control groups after treatment

	Description	Control group (n = 207)	Experimental group (n = 205)	Statistics (Z)	P-value
Fever relief time (h)	Median	48.00	48.00	0.174	0.862
	P25 ~ P75	48.00 ~ 72.00	48.00 ~ 72.00		
	Min-max	24 ~ 144	24 ~ 144		
Influenza Symptom relief time (h)	median	60.00	48.00	1.467	0.142
	P25 ~ P75	42.00 ~ 72.00	24.00 ~ 72.00		
	Min-max	24 ~ 216	24 ~ 120		

#### Severity of influenza symptoms

**Fig. 4:**
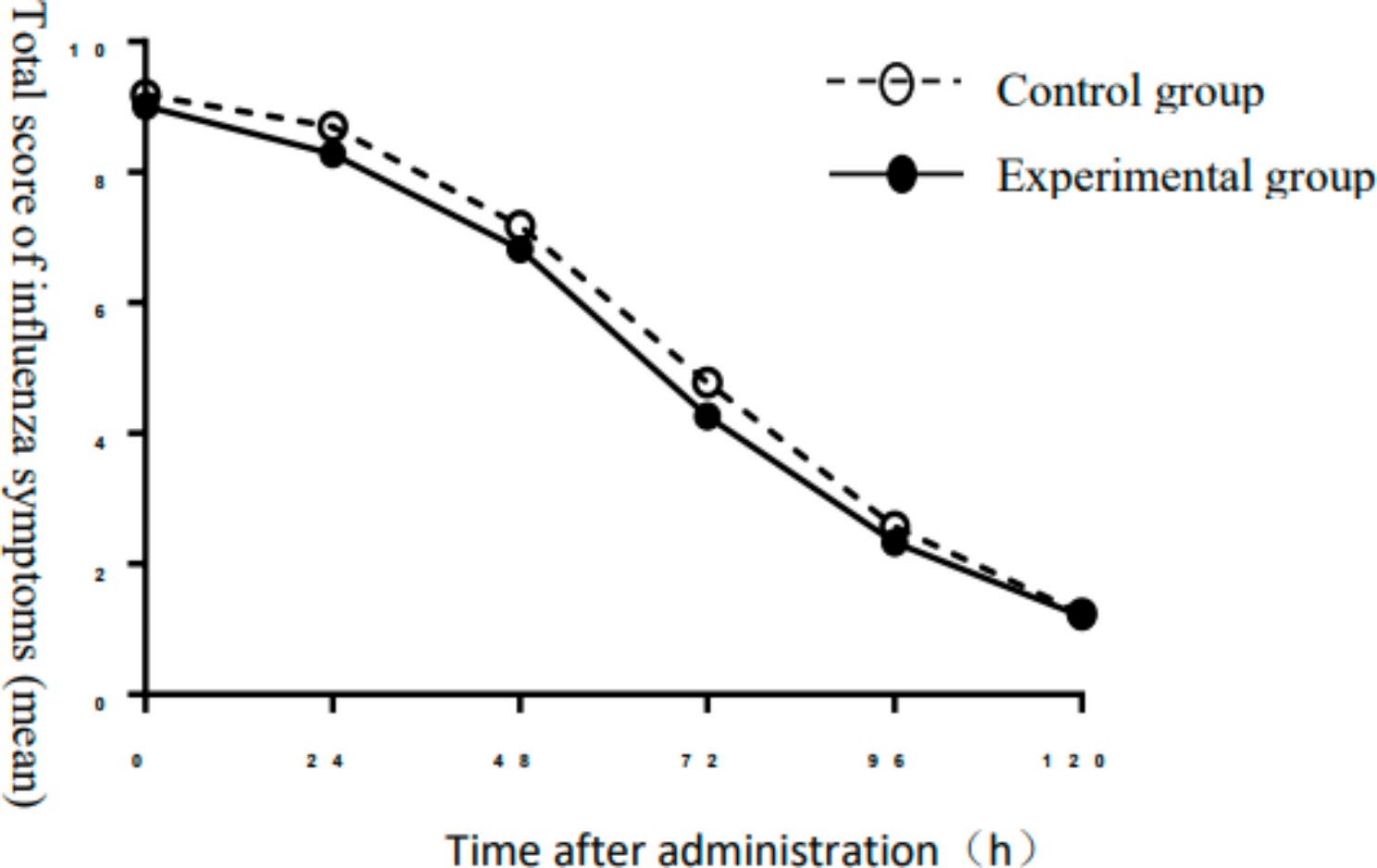
Total score of influenza symptoms – AUC over time

The AUC values of the control and experimental groups were 682.6 ± 141.9 and 643.1 ± 148.1, respectively, and there was no significant difference between the two groups (*P* > 0.05) (Fig. 4).

### Safety evaluation

Regarding complications, there was only one case of tracheitis in the control group. For adverse events, there was one case of nausea, one case of vomiting, and one case of dizziness in the control group; in the experimental group, there was one case of nausea, one case of vomiting, and one case of drowsiness. In addition, one patient in the control group was hospitalized because of urinary calculi. Single symptoms of adverse events were difficult to compare because of the small number of cases.

## Discussion

The neuraminidase inhibitor oseltamivir selectively inhibits the activity of neuraminidase on the surface of respiratory tract viruses, preventing the reproduction and release of progeny virus particles in human cells and resulting in a therapeutic effect on influenza [18, 19]. At present, oseltamivir is the gold standard and the most widely used anti-influenza drug, with proven efficacy against influenza A and B infections [3–5]. As drug-resistant variants continue to emerge naturally through selective pressure caused by antiviral drug use, the efficacy of oseltamivir may wane over time [6–9]. Therefore, new influenza therapeutics with novel mechanisms of action against new targets are urgently required to combat the persistent threat of influenza viruses. Some studies have demonstrated that oseltamivir-resistant viruses are susceptible to arbidol [13, 14], suggesting that it may be a good alternative for the clinical treatment of infections caused by oseltamivir-resistant viruses.

Influenza viruses can share RNA segments that develop into new generations of strains and different subtypes [20]. Major antigenic mutations arise from viruses that merge their surface antigens, hemagglutinin (HA) and neuraminidase (NA), from two or more original strains to create a new strain [21]. The broad-spectrum antiviral drug arbidol [22–25] is effective against influenza viruses by targeting the HA fusion machinery. Arbidol can interact with the viral protein HA in both influenza virus A and B. It binds to a hydrophobic cavity in the HA trimer stem at the interface between two monomers, which leads to the stabilization of the prefusion conformation of HA.

Arbidol has been approved in several countries for prophylaxis and treatment of influenza [24, 26, 27]. Observational studies are important additional tools that should be considered when estimating the effectiveness of antiviral therapy. We conducted a prospective, multicenter, randomized controlled trial to evaluate the clinical efficacy of arbidol in the treatment of influenza-like cases. This randomized trial found no difference in efficacy between the two groups in time to return to normal temperature and relief of influenza symptoms. Clinical tests for the prophylactic and therapeutic properties of arbidol with respect to both influenza and respiratory viral infections of noninfluenzal etiology have been conducted for several years and involved about 8 thousand adults and more than 500 children, including infants aged above 6 months. The results indicated that arbidol had a remarkable effect on prevention and treatment [28]. This was consistent with the results of our study. This suggests that arbidol is equivalent to oseltamivir in improving the clinical symptoms of influenza in influenza-like cases. Another observational study estimated the clinical effectiveness of oseltamivir and arbidol during an influenza season in Russia [13], and the results were similar to those of this study.

In this study, we found that arbidol was safe and well-tolerated, and only a few minor events, such as nausea, vomiting, and drowsiness, were reported. No deaths were reported in either arm. This is similar to the reports of minor adverse events in other trials involving arbidol. It is noteworthy that arbidol has been used clinically for decades in other countries, with minimal side effects and a good pharmacokinetic profile [24, 26]. Clinical tests for the prophylactic and therapeutic properties of arbidol with respect to both influenza and respiratory viral infections of noninfluenzal etiology have been conducted for several years and involved about 8 thousand adults and more than 500 children, including infants aged above 6 months. The results showed that arbidol was well-tolerated and had no side effects [28]. Moreover, arbidol has been used for decades in China and Russia to treat influenza and other respiratory viral infections, with no major adverse effects [26]. Arbidol interacts with viral HA to inhibit its function [29] and has been shown to work against oseltamivir-resistant viruses [13, 30]. However, despite years of over-the-counter use in China and Russia to treat influenza, arbidol-resistant mutations have yet to be reported [26].

In addition to exerting antiviral and anti-inflammatory activities against various types of influenza viruses [31, 32], arbidol also exhibits broad-spectrum antiviral activities against other viruses, including DNA and RNA viruses, as well as capsid- and membrane-enclosed viruses [22–24, 33], such as respiratory syncytial virus, hepatitis B virus, adenovirus, and Hantaan virus [10, 34]. It thus has vast potential as a broad-spectrum antiviral agent, as indicated by in vitro and in vivo studies [10, 27, 34–37], lending hope for its clinical use against various infectious diseases that are currently not therapeutically controlled. We are currently living in an unprecedented crisis, and arbidol is currently undergoing clinical trials against COVID-19 [38, 39]. Owing to its broad-spectrum antiviral activities, arbidol is a promising candidate for the treatment of viral infections in humans. In particular, at present, timely diagnosis of influenza is still difficult and requires expensive diagnostic methods, and given the need to start treatment as early as possible, patients that meet the criteria used herein can benefit from treatment with arbidol without waiting for virological results during epidemics.

### Limitations

The limitations of the study are as follows: there was no etiological virus typing and antiviral drug sensitivity detection. Moreover, children, the elderly, and patients with serious pre-existing medical conditions who tend to be more susceptible to influenza and complications due to low resistance were excluded. Additionally, the study was conducted in only one province due to regional limitations, and thus the results cannot be generalized. We hope to expand the study scale and number of cases in the future to further explore the effect of early treatment with arbidol tablets on the outcome, prognosis, and spread of suspected influenza cases in China.

## Conclusion

There is no significant difference between arbidol hydrochloride tablets and oseltamivir phosphate capsules in the treatment of influenza-like cases, and arbidol hydrochloride tablets have less adverse reactions and are safe to use. The early use of arbidol hydrochloride after the onset of influenza-like cases can shorten the duration of disease and reduce the severity of symptoms, and its safety and tolerance are good, and thus is suitable for clinical promotion.

## Data Availability

The original contributions presented in the study are included in the article, further inquiries can be directed to the corresponding author.

## Declarations

### Accordance Statement

The clinical research was carried out in accordance with the Helsinki Declaration of the World Medical Congress, the Good Clinical Practice and other relevant regulations.
